# Supplementary material for: Mapping the microRNA-mediated crosstalk between insulin resistance and Alzheimer’s disease: A computational genomic insight
Source: PLoS One. 2025 Dec 12;20(12):e0329056. doi: 10.1371/journal.pone.0329056 (PMC12700384; doi:10.1371/journal.pone.0329056)
Supplement: S1 File — (DOCX) [file pone.0329056.s001.docx]

# Supplementary File S1. Summary of genes included and excluded from the insulin resistance pathway analysis

| **Category** | **Genes** | **Function** | **Rationale for Inclusion / Exclusion** | **Ref** |
| --- | --- | --- | --- | --- |
| **INCLUDED*** |  |  |  |  |
| Upstream regulators (signal initiation) | INSR, IRS1, IRS2, PIK3R1, PIK3R2, AKT2, AKT3 | Initiate insulin signaling: INSR autophosphorylation activates IRS1/2, which recruit PI3K (via PIK3R1/2) to generate PIP3 and activate AKT kinases (AKT2/3). | Represent the canonical PI3K–AKT metabolic signaling pathway. Dysregulation at these nodes is central to insulin resistance. | (1-3) |
| Downstream regulators (signal propagation and integration) | GSK3B, PDK1, FOXO1, MTOR, TSC1, TSC2, AKT1S1 (PRAS40) | Mediate downstream metabolic actions of insulin through the PI3K–AKT–mTOR axis. | Represent canonical AKT substrates and mTORC1 regulators that coordinate glucose utilization, glycogen synthesis, and anabolic signaling. | (4, 5) |
| Terminal metabolic effectors | ACACA, ACACB, PDE3B, ABHD15, SLC2A4 (GLUT4) | Execute insulin’s key metabolic effects. | Capture the final effector layer of insulin signaling: SREBF1 and ACACA/B for lipogenesis, PDE3B and ABHD15 for suppression of lipolysis, and SLC2A4 for insulin-stimulated glucose transport. | (6-8) |
| **EXCLUDED^** |  |  |  |  |
| Mitogenic arm components | GRB2, SHC, SH2B2 | Adaptors linking INSR to the RAS–MAPK mitogenic pathway or CAP–Cbl signaling in specific tissues. | The study focuses on the metabolic arm (PI3K–AKT) of insulin signaling; mitogenic and tissue-specific pathways were outside scope. | (3, 9) |
| Upstream and feedback modulators | PTP1B (PTPN1), SOCS1, SOCS3, PTEN, SHIP2 (INPPL1) | Phosphatases and suppressors that attenuate insulin signaling via dephosphorylation or feedback inhibition. | Excluded as they act via negative regulation rather than direct propagation of insulin signaling. |  |
| Inflammatory and transcriptional modulators | IL6, TNF, TLR4, PPARG, AMPK (PRKAA1/2) | Cytokines and transcriptional regulators that affect insulin sensitivity through inflammation or lipid metabolism. | Excluded because they modulate insulin sensitivity indirectly, not as core components of the INSR–IRS–PI3K–AKT–mTOR signaling cascade. |  |
| Other glucose transporters | SLC2A2 (GLUT2) | Low-affinity glucose transporter expressed in liver and pancreatic β-cells. | Excluded because it mediates glucose sensing, not insulin-stimulated glucose uptake. SLC2A4 (GLUT4) was retained as the canonical insulin-responsive transporter. |  |

*Included genes represent the canonical PI3K–AKT–mTOR metabolic signaling cascade and its downstream metabolic effectors.
^Excluded genes include mitogenic, feedback, and inflammatory regulators outside this defined scope.

**References**

1. Pei J, Wang B, Wang D. Current Studies on Molecular Mechanisms of Insulin Resistance. J Diabetes Res. 2022;2022:1863429. doi: 10.1155/2022/1863429.

2. Chandrasekaran P, Weiskirchen R. Cellular and Molecular Mechanisms of Insulin Resistance. Curr Tissue Microenviron Rep. 2024;5(3):79-90. doi: 10.1007/s43152-024-00056-3.

3. Le TKC, Dao XD, Nguyen DV, Luu DH, Bui TMH, Le TH, et al. Insulin signaling and its application. Front Endocrinol (Lausanne). 2023;14:1226655. doi: 10.3389/fendo.2023.1226655.

4. Huang X, Liu G, Guo J, Su Z. The PI3K/AKT pathway in obesity and type 2 diabetes. Int J Biol Sci. 2018;14(11):1483-96. doi: 10.7150/ijbs.27173.

5. Taheri R, Mokhtari Y, Yousefi AM, Bashash D. The PI3K/Akt signaling axis and type 2 diabetes mellitus (T2DM): From mechanistic insights into possible therapeutic targets. Cell Biol Int. 2024;48(8):1049-68. doi: 10.1002/cbin.12189.

6. Xia W, Pessentheiner AR, Hofer DC, Amor M, Schreiber R, Schoiswohl G, et al. Loss of ABHD15 Impairs the Anti-lipolytic Action of Insulin by Altering PDE3B Stability and Contributes to Insulin Resistance. Cell Rep. 2018;23(7):1948-61. doi: 10.1016/j.celrep.2018.04.055.

7. Wang T, Wang J, Hu X, Huang XJ, Chen GX. Current understanding of glucose transporter 4 expression and functional mechanisms. World J Biol Chem. 2020;11(3):76-98. doi: 10.4331/wjbc.v11.i3.76.

8. Ferré P, Phan F, Foufelle F. SREBP-1c and lipogenesis in the liver: an update1. Biochem J. 2021;478(20):3723-39. doi: 10.1042/bcj20210071.

9. Desbuquois B, Carré N, Burnol AF. Regulation of insulin and type 1 insulin-like growth factor signaling and action by the Grb10/14 and SH2B1/B2 adaptor proteins. Febs j. 2013;280(3):794-816. doi: 10.1111/febs.12080.
